# Investigating factors influencing overweight and obesity among adult households in Ethiopia: a multilevel ordered analysis of 2016 EDHS data

**DOI:** 10.3389/fendo.2024.1408090

**Published:** 2024-10-14

**Authors:** Alemayehu Deressa, Dawit Firdisa, Abdi Birhanu, Adera Debella, Mulugeta Gamachu, Addis Eyeberu, Deribe Bekele Dechasa, Usmael Jibro, Bikila Balis, Moti Tolera, Lemma Demissie Regassa, Ibsa Mussa

**Affiliations:** ^1^ School of Public Health, College of Health and Medical Sciences, Haramaya University, Harar, Ethiopia; ^2^ School of Medicine, College of Health and Medical Sciences, Haramaya University, Harar, Ethiopia; ^3^ School of Nursing and Midwifery, College of Health and Medical Sciences, Haramaya University, Harar, Ethiopia; ^4^ Department of Public Health, Rift Valley University, Harar, Ethiopia

**Keywords:** overweight, obesity, body mass index, adult, Ethiopia

## Abstract

**Background:**

In both high- and low-income countries, including Ethiopia, overweight and obesity have emerged as public health issues of the 21st century. Hence, obtaining conclusive evidence concerning the factors that influence adults’ body mass index is important. Therefore, using representative data, our study sought to provide solid evidence on factors influencing overweight and obesity among adults in Ethiopia.

**Methods:**

The 2016 Ethiopia Demographic and Health Survey (EDHS), a dataset composed of a nationally representative sample of the survey, served as the basis for the study. Both descriptive and analytic findings were produced using STATA version 14. The data collection were conducted from January to June 2016. A total sample of 39,749 adults, 18 years and older, were included. Predictors were assessed using multivariable ordinal logistic regression analysis, and the results were presented as an adjusted proportional ratio (POR) with a 95% confidence interval. Statistical significance was declared at a *p*-value of <0.05.

**Results:**

Overall, the magnitude of overweight and obesity among adults in Ethiopia was 8.5% (95% CI: 8.2% to 8.7%) and 2.9% (95% CI: 2.7% to 3.1%), respectively. Predictor variables such as smoking (POR = 0.53, 95% CI: 0.42–0.67); being female (POR = 1.21, 95% CI: 1.13–1.30); being married (POR = 1.91, 95% CI: 1.26–2.90); having a secondary education (POR = 1.42, 95% CI: 2.23–1.64); having a diploma and above education (POR = 1.78, 95% CI: 1.44–2.21); having a poorer (POR = 1.22, 95% CI: 1.13–1.31), middle (POR = 1.30, 95% CI: 1.20–1.40), richer (POR = 1.35, 95% CI: 1.25–1.47), and richest (POR = 3.13, 95% CI: 2.79–3.51) wealth index rating; and having a rural residence (POR = 0.48, 95% CI: 0.43–0.54) were significantly associated with overweight and obesity.

**Conclusions:**

Overall, 8.5% and 2.9% of Ethiopian adults were overweight and obese, respectively. Factors such as smoking, sex, marital status, educational status, wealth index, residence, and region were significantly associated with overweight and obesity among adult households. As a result, enhancing lifestyle modifications is enormous, and it is necessary to have more tangible evidence concerning the factors influencing body mass index utilizing more representative data from local and global.

## Introduction

Overweight and obesity are defined as abnormal or excessive fat accumulation that may impair the health of individuals (1). Body mass index (BMI) is a simple index of weight for height that is commonly used to classify overweight and obesity in adults and also stipulates the most useful population-level measure of overweight and obesity as it is the same for both sexes and for all ages (2, 3). A high BMI can designate high body fatness and is used for screens for weight categories that may lead to health problems; its range is differently categorized: Its range is divided into four categories: underweight (BMI less than 18.5), healthy weight (18.5 to 24.9), overweight (25.0 to 29.9), and obese (30.0 or higher) (4).

Currently, overweight and obesity are public health concerns, with obesity being one of the leading risk factors for premature death and 8% of global deaths being ascribed to obesity (5, 6). In most high-income countries, approximately two-thirds of adults are classified as overweight or obese; compared to the regions of South Asia and Sub-Saharan Africa, where the rate is closer, around one in five adults have a BMI greater than 25 (5). In addition, both obesity and overweight are associated with amplified disease-specific morbidity, 4.0 million deaths globally, and approximately 36.3 million YLLs, and cost a total of $4.32 trillion worldwide, $3.8 billion in health expenditure in Saudi Arabia, and $552.31 million in Ethiopia (7–12).

Factors related to the problem of overweight and obesity are unhealthy lifestyle habits, such as a sedentary lifestyle, smoking, and eating high-calorie, low-nutrient foods and beverages. Additionally, socioeconomic and health conditions can raise the overweight and obesity levels of individuals (13–16). Therefore, limiting unhealthy foods (refined grains and sweets, potatoes, and processed meat) and beverages (sugary drinks); increasing physical activity; limiting television, screen time, and other “sit time”; improving sleep; and reducing stress are all recommended as ways to prevent, treat, and control the health consequences of being overweight and obese (17, 18).

A lot of effort has been made to alleviate the burden of overweight and obesity. The World Health Organization (WHO) calls on all stakeholders to take action at the global, regional, and local levels to improve diets and physical activity patterns at the population level and recognizes that non-communicable diseases (NCDs) are a major challenge for sustainable development goals in the 2030 Agenda for Sustainable Development (19). The Ethiopian government has worked on the prevention and control of adult overweight and obesity through encouraging physical activity, promoting fruit intake and whole grain intake, educating on reducing processed meat intake, and providing mental health support (11). Even though many efforts were made on reducing overweight and obesity, the magnitude and morbidity of overweight and obesity are still increasing in developed and developing countries, including Ethiopia (20–22).

Overweight and obesity are complex issues caused by genetic, behavioral, cultural, and environmental factors. Changes in policies within the government, social environment, and cultural norms are required, in addition to individual behavioral changes. Most nutritional programs in Ethiopia focus on childhood undernutrition. Identifying factors influencing overweight and obesity is crucial for preventing and managing these developing public health issues in Ethiopia (23). Therefore, this study was conducted to further investigate the factors influencing overweight and obesity among adults in Ethiopia using representative data.

## Methods and materials

### Study design, setting, and period

The source of this study is the nationally representative 2016 Ethiopia Demographic and Health Survey (EDHS). Ethiopia is located between 3° and 15° North latitudes and 33° and 48° East longitudes. The country has nine regional states [Amhara, Oromia, Tigray, Benishangul-Gumuz, Somali, Afar, Harari, Southern Nations Nationalities and Peoples (SNNP), and Gambella] and two city administrations (Addis Ababa and Dire Dawa) (24, 25). The survey was conducted in a nationally representative sample and provided estimates at national and regional levels for rural and urban areas.

### Population and sampling

The EDHS used a two-stage cluster sampling design with rural–urban regions as strata. In the first stage, enumeration areas (EAs) were selected with probability proportional to EA size, with independent selection in each sampling stratum. In the second stage, an appropriate number of households per cluster were selected with an equal probability of systematic selection. This study used a total sample of 39,749 adults in the analysis that was extracted from the EDHS survey. Further information regarding the sampling technique and questionnaire, in general about the survey, can be accessed from the EDHS 2016 report (26). Datasets for adults were downloaded from the MEASURE DHS website.

### Study variables and measurements

The outcome variables were as follows: underweight, normal, overweight, and obesity, based on the BMI measurement of adult people. Overweight and obesity are categorized based on the WHO Classification of BMI: BMI = weight in kilograms divided by height in meters squared. Individuals were classified as overweight if their BMI is 25.0–29.9 kg/m^2^ and as obese if their BMI is 30 kg/m^2^ or higher (27). The independent variables included demographic characteristics and behavior-related characteristics of adult people. In addition, the community-level factors like region and residence were considered as explanatory variables.

### Statistical analysis

Frequency distribution analysis was carried out. Data were analyzed using STATA version 14 statistical software. The data were weighted using sampling weight, primary sampling unit, and strata before any statistical analysis to ensure the representativeness of the survey and to consider the sampling design when calculating standard errors to get reliable statistical estimates.

To determine the relationship between household wealth index and overweight and obesity as well as to control confounders, logistic regression assumptions (chi-square and multicollinearity) were tested. Because members in a cluster shared a trait, the assumptions of the independence of observations and equal variance across clusters were broken. To ensure the accuracy of the standard error and unbiased estimate, it is important to overcome the violated independence assumption and take into account the variability between clusters in multilevel advanced statistical modeling.

Variables having a *p*-value of 0.25 were candidates for the individual level (Model II), community level (Model III), and the final model (Model IV). The mixed-effect model with the lowest Akaike and Bayesian Information Criteria (AIC and BIC) was chosen. A factor was designated as a significant predictor of obesity and overweight if its *p*-value was less than 0.05. The odds ratio was used to measure how strong the link was, with a 95% confidence interval. The estimation of the association between the BMI (underweight, normal, overweight, and obesity) and explanatory variables was performed using the fixed-effects model. An intra-cluster correlation coefficient (ICC) with standard deviation was employed to measure cluster variance. A null model (a model without independent variables), a model taking into account only individual-level factors, a model taking into account community-level variables, and a fourth model taking into account both individual and community-level variables were also fitted. The comparison of the multilevel-ordered logistic regression models was checked using the median odds ratio (MOR) and proportional change in variance (PCV). The best-fitting model among the fitted models was ultimately chosen using the AIC and BIC.

## Results

### Community-level, household, and individual-related characteristics of the study participants

Out of a total of 41,357 adults, 39,749 were included in this study. Approximately 5,788 (14.56%), 5,346 (13.5%), 4,888 (12.3%), 4,604 (11.58%), and 4,358 (10.96%) adults were included from the Oromia, SNNPR, Somalia, Amhara, and Tigray regions, respectively. More than two-thirds [*n* = 32,581 (81.97%)] of the study participants resided in rural areas. The majority (*n* = 29,052 adults) had no formal education. The majority of the study participants were men. Nearly one-third of the participants had the poorest wealth index. About *n* = 38,046 (95.7%) had no health insurance coverage. The majority of the participants [*n* = 20,872 (52.5%)] were not working (**Table 1**).

**Table 1:** Community-level, household, and individual-related characteristics of the study participants.

Variables	Frequency	Percent
Region		
Tigray	4,358	10.96
Afar	3,349	8.43
Amhara	4,604	11.58
Oromia	5,788	14.56
Somali	4,888	12.3
Benishangul	3,370	8.48
SNNPR	5,346	13.45
Gambela	2,515	6.33
Harari	1,845	4.64
Addis Ababa	1,596	4.02
Dire Dawa	2,090	5.26
Place of residence		
Urban	7,168	18.03
Rural	32,581	81.97
Educational status		
No formal education	29,052	73.09
Primary	8,043	20.23
Secondary	1,791	4.51
Higher	863	2.17
Religion		
Orthodox	13,173	33.14
Catholic	206	0.52
Protestant	7,252	18.24
Muslin	18,547	46.66
Traditional	336	0.85
Other	235	0.59
Sex of the participant		
Male	29,910	75.25
Female	9,839	24.75
Age of the respondent		
35 and below	37,912	95.38
Above 35 years	1,837	4.62
Wealth index of the household		
Poorest	13,583	34.17
Poorer	6,548	16.47
Middle	5,846	14.71
Richer	5,851	14.72
Richest	7,921	19.93
Covered by health insurance		
No	38,046	95.72
Yes	1,703	4.28
Occupational status		
Not working	20,872	52.51
Professional/technical/managerial	572	1.44
Clerical	176	0.44
Sales	5,279	13.28
Agricultural—employee	9,392	23.63
Services	710	1.79
Skilled manual	1,391	3.5
Unskilled manual	667	1.68
Others	690	1.74

### Behavioral characteristics of the study participants

Among the total adults, nearly one-third [*n* = 11,972 (30.1%)] of the study participants had a history of alcohol consumption. A total of 513 (1.3%) study participants were smokers. A total of 4,940 (12.3%) adults had a history of chewing khat. More than half [*n* = 26,546 (66.78%)] were not working at the time of data collection. Nearly two-thirds of the *n* = 26,947 (67.8%) study subjects were sexually active 4 weeks before the data collection time (**Table 2**).

**Table 2:** Behavioral characteristics of the study participants.

Variables	Frequency	Percent
Alcohol consumption		
No	27,777	69.88
Yes	11,972	30.12
Smoking status		
No	39,236	98.71
Yes	513	1.29
Sexually active		
Active in the last 4weeks	26,947	67.79
Not active due to postpartum	3,142	7.9
Not active	9,660	24.3
Currently working		
No	26,546	66.78
Yes	13,203	33.22
Khat chewing		
No	34,809	87.57
Yes	4,940	12.43

### The magnitude of BMI classifications

**Figure 1:**
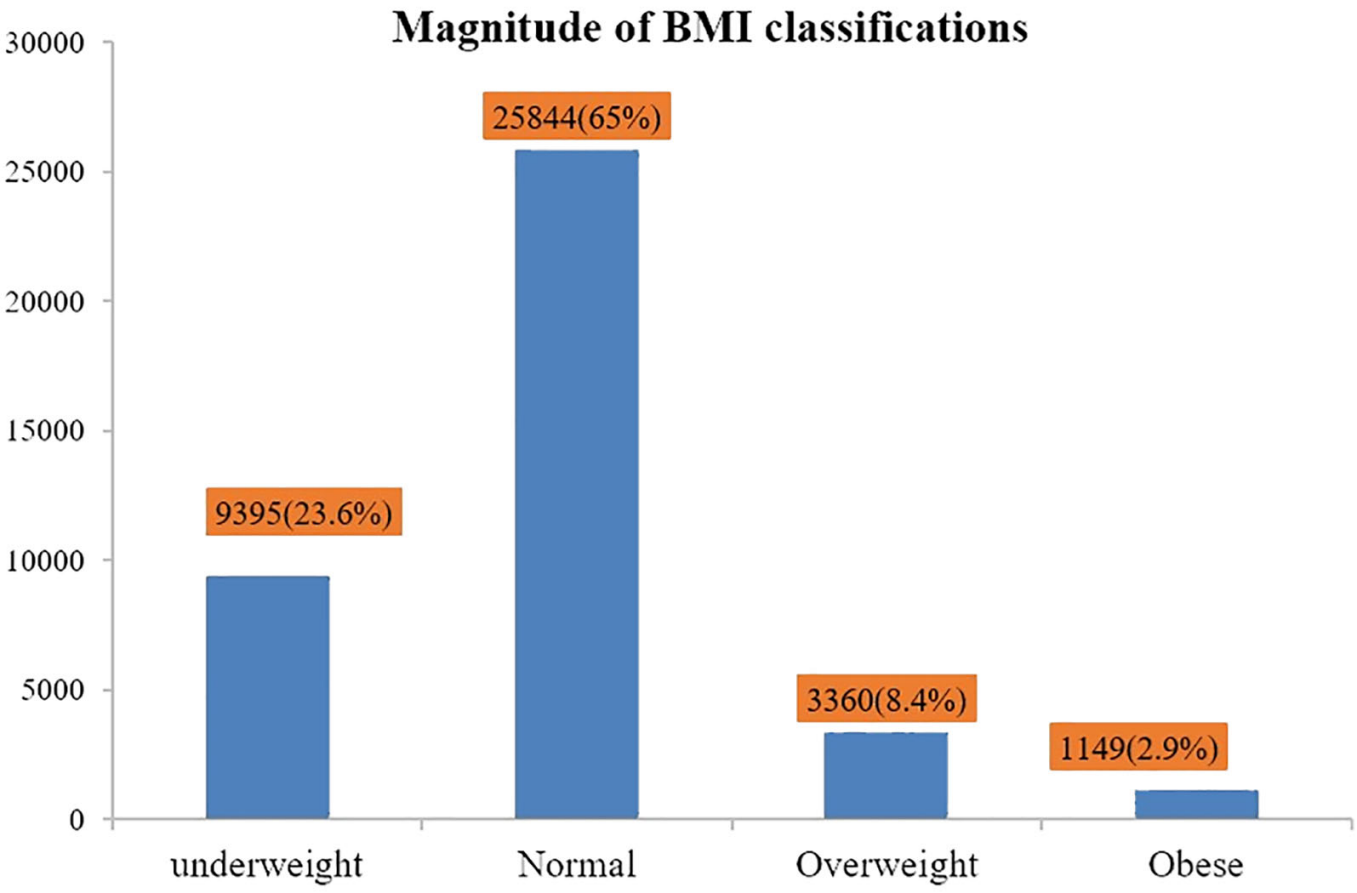
Magnitude of outcome variables (underweight, normal, overweight, and obesity).

Out of the total *n* = 39,749 Ethiopian adult populations, *n* = 9,395 (23.6%), *n* = 25,844 (65%), *n* = 3,360 (8.4%), and *n* = 1,149 (2.95%) had underweight, normal, overweight, and obese BMI status, respectively (**Figure 1**).

### Distribution of BMI classification among the study participants

Almost a quarter [*n* = 1,743 (24.3%)] of rural residents were overweight. Additionally, *n* = 1,103 (11.2%) of women were also overweight. Almost 1 in 10 [*n* = 2,503 (9.0%)] adults over the age of 35 were overweight. Furthermore, 1 out of every 10 [*n* = 871 (11.0%)] richest adults was obese. Of the *n* = 513 smokers, 30 (5.8%) were obese (**Table 3**).

**Table 3:** The distribution of BMI classification and relationships with factors among study participants.

Variable	Category	Underweight	Normal	Overweight	Obese	Total	Chi *p*-value
Place of residence	Urban	782	3,815	1,743	828	7,168	0.000
	Rural	8,614	22,029	1,617	321	32,581	
Sex	Male	7,059	19,889	2,257	705	29,910	0.000
	Female	2,337	5,955	1,103	444	9,839	
Age in years	At least 35	2,684	7,937	857	308	11,786	0.000
	Greater than 35	6,712	17,907	2,503	841	27,963	
Educational status	No education	7,354	19,424	1,801	473	29,052	0.000
	Primary	1,667	5,180	866	330	8,043	
	Secondary	282	880	403	226	1,791	
	Higher	93	360	290	120	863	
Wealth index	Poorest	4,454	8,312	700	117	13,583	0.000
	Poorer	1,537	4,714	236	61	6,548	
	Middle	1,318	4,234	241	53	5,846	
	Richer	1,247	4,220	337	47	5,851	
	Richest	840	4,364	1,846	871	7,921	
Marital status	Never in union	36	110	13	14	173	0.000
	Married	8,202	23,022	2,845	956	35,025	
	Living with partner	94	275	34	13	416	
	Widowed	536	1,122	219	65	1,942	
	Divorced	418	1,046	191	78	1,733	
	No longer living together	110	269	58	23	460	
Smoking status	No	9,206	25,562	3,330	1,138	39,236	0.000
	Yes	190	282	30	11	513	
Alcohol consumption status	No	6,860	17,719	2,387	811	27,777	0.000
	Yes	2,536	8,125	973	338	11,972	
Chewing khat	No	8,326	22,697	2,864	922	34,809	0.000
	Yes	1,070	3,147	496	227	4,940	

### Factors associated with overweight and obesity

The bivariable analysis revealed that variables having a *p*-value less than 0.2 were transformed into multilevel multivariable ordinal logistic regression. Finally, it resulted in significant associations between predictor variables, including smoking, sex, marital status, educational status, wealth index, residence, and region, and the dependent variable (overweight and obesity) (**Table 4**). Accordingly, those adults who had a habit of smoking cigarettes were 47% less likely to be overweight or obese as compared to adults who did not smoke (POR = 0.53; 95% CI: 0.42–0.67). In addition, those adults who reside in rural areas were 52% less likely to be overweight or obese than their counterparts (POR = 0.48, 95% CI: 0.43–0.54).

**Table 4:** A multilevel analysis of overweight and obesity determining factors.

Variable	Overweight/Obesity	Model I (null)	Model II (individual level)	Model III (community level)	Model IV (AOR 95% CI)
Region					
Tigray	291			1	1
Afar	245			0.64 (0.46–0.90)	1.1 (0.94–1.24)
Amhara	148			1.37 (1.00–1.87)	1.31 (1.19–1.44)
Oromia	408			1.44 (1.06–1.97	1.65 (1.48–1.84)
Somali	982			2.23 (1.62–3.07)	3.04 (2.64–3.49)
Benishangul	195			1.75 (1.24–1.48)	2.03 (1.81–2.28
SNNPR	360			1.89 (1.38–2.59)	2.16 (1.93–2.42)
Gambela	155			0.55 (0.39–0.78)	0.78 (0.68–0.91
Harari	389			2.28 (1.57–3.03)	1.95 (1.65–2.29)
Addis Ababa	810			3.77 (2.59–5.48)	3.56 (3.00–4.23)
Dire Dawa	526			2.28 (1.58–3.32)	2.16 (1.77–2.41)
Place of residence					
Urban	2,571			1	1
Rural	1,938			0.15 (0.12–0.19)	0.49 (0.44–0.55)
Household wealth index					
Poorest	817		1		1
Poorer	297		1.27 (1.18–1.37)		1.19 (1.10–1.28)
Middle	294		1.31 (1.20–1.43)		1.26 (1.16–1.36)
Richer	384		1.41 (1.29–1.59)		1.32 (1.22–1.43)
Richest	2,717		3.20 (2.85–3.60)		2.94 (2.61–3.31)
Educational status					
No education	2,274	1	1		
Primary	1,196		1.09 (1.02–1.16)		1.01 (0.95–1.12)
Secondary	629		1.60 (1.43–1.82)		1.44 (1.24–1.68)
Higher	410		1.86 (1.57–2.19)		1.79 (1.43–2.23)
Religion					
Orthodox	1,691				
Catholic	12		0.72 (0.52–1.01)		0.60 (0.42–0.85)
Protestant	519		0.83 (0.74–0.94)		0.71 (0.64–0.77)
Muslim	2,264		0.81 (0.72–0.91)		0.76 (0.70–0.82)
Traditional	9		1.03 (0.75–1.41)		1.02 (0.82–1.25)
Other	14		0.78 (0.56–1.09)		1.28 (1.01–1.64)
Sex					
Male	2,962		1		1
Female	1,547		1.13 (1.06–1.21)		1.18 (1.10–1.27)
Age of the participant	Mean = 42.57 (SD = +7.3)		1.01 (1.00–1.04)		1.00 (0.99–1.01)
Covered by health insurance					
No	4,358		1		1
Yes	151		1.04 (0.91–1.19)		0.91 (0.81–1.02)
Marital status					
Single	27		1		
Married	3,801		2.10 (1.49–2.94)		1.82 (1.20–2.77)
Living with partner	47		1.82 (1.22–2.71)		1.19 (0.74–1.91)
Widowed	284		1.67 (1.18–2.13)		1.34 (0.87–2.06)
Divorced	269		1.41 (0.99–2.00)		1.20 (0.78–1.85)
Living together/separate	81		1.63 (1.10–2.41)		1.45 (0.89–2.36)
Smoking cigarettes					
No	4,468		1		
Yes	41		0.57 (0.46–0.70)		0.38 (0.19–0.77)
Alcohol consumption					
No	3,198		1		
Yes	1,311		0.88 (0.81–0.96)		1.02 (0.96–1.11)
Khat chewing					
No	3,786		1		1
Yes	723		1.35 (1.23–1.47)		1.15 (0.97–1.16)
Variance		2.05 (0.12)	1.16 (0.07)	0.77 (0.05)	0.69 (0.04)
LR		9,014.23	4,805.99	4,090.82	3,418.91
AIC		65,095.61 (df = 4)	64,459.64 (df = 27)	64,541.77 (df = 15)	64,127.33 (df = 38)
Model comparison					
MORE			2.78	2.30	2.20
PCV			1.48	1.67	1.71

In another way, adult households with a wealth index of poorer, middle, richer, and richest were 1.22, 1.30, 1.35, and 3.13 times more likely to be overweight or obese compared to adult households with a wealth index of poorest (POR = 1.22, 95% CI: 1.13–1.31; 1.30, 95% CI: 1.20–1.40; 1.35, 95% CI: 1.25–1.47; and 3.13, 95% CI: 2.79–3.51), respectively. Moreover, adults whose educational status was secondary and diploma and above were 1.42 and 1.78 times more likely to be overweight or obese compared to adult households whose educational status is no formal education (POR = 1.42, 95% CI: 2.23–1.64 and 1.78, 95% CI: 1.44–2.21), respectively. Furthermore, female adults were 1.21 times more likely to be overweight or obese compared to adult households who were male (POR = 1.21, 95% CI: 1.13–1.30). Finally, the study revealed that the odds of being overweight or obese were 1.95 times higher among married adults than unmarried adults (POR = 1.91, 95% CI: 1.26–2.90) (**Table 4**).

## Discussion

This study was conducted to determine the factors affecting overweight and obesity among adults in Ethiopia using representative data. According to this study, the overall magnitude of overweight and obesity among adult residents in Ethiopia was 8.5% (95% CI: 8.2% to 8.7%) and 2.9% (95% CI: 2.7% to 3.1%), respectively. The final model of multivariable ordinal logistic regression analysis indicated that predictor variables including smoking, sex, marital status, educational status, wealth index, residence, and region were significantly associated with overweight and obesity among adult households. The study revealed that, among overweight and obese adults, approximately 1.0% (95% CI: 0.6% to 1.2%) of them were cigarette smokers.

While some studies have shown no significant association between smoking status and obesity or overweight, others have suggested that smoking may significantly affect obesity or overweight (28–30). Our final model of multivariable analysis explicitly pointed out that adults’ habit of cigarette smoking was found to be associated with obesity/overweight. Accordingly, those adults who had a habit of cigarette smoking were 47% less likely to develop overweight or obesity as compared to adults who did not smoke. This finding is consistent with a study carried out in different regions of the world to determine the association between smoking and overweight or obesity (29, 31–33). One plausible explanation related to these facts could be that those who smoke cigarettes are less likely to have physical exercise, which inevitably leads those adults to experience overweight or obesity (29, 34–36).

The study also revealed that adult households with a wealth index of poorer, middle, richer, and richest had 1.22, 1.30, 1.35, and 3.13 times more likely to be overweight or obese compared to adult households with a wealth index of poorest. This finding is in line with similar studies that indicate that as the wealth index tertile rises, adults are more likely to be overweight or obese (37–39). This is explicitly related to the fact that as people’s income rises, sedentary lifestyles, such as traveling by automobile and eating a nutritious diet, may become more common at the individual or household levels; these activities increase the likelihood of developing overweight and obesity (40, 41). On this occasion, even though they are busy managing their wealth, they have the ability to consume the kind of food they need, and move from one place to another by car, and adults in this situation still need to think about healthy eating, physical activity, maintaining a healthy weight, managing stress, and getting enough quality sleep.

The study also depicted that adults whose educational status was secondary and diploma and above were 1.42 and 1.78 times more likely to be overweight or obese compared to adult households whose educational status is no formal education, respectively. This study is consistent with other research showing the relationship between educational attainment and the likelihood of being overweight or obese (42–44). One possible explanation could be that as educational attainment rises, the likelihood of earning a high income and residing in an urban area also rises. As a result, living in an urban area and earning a high income may expose adults to a sedentary lifestyle such as moving place to place by transportation, as well as increase the probability of consuming high-calorie food and drinks (45, 46).

In addition, our study revealed that female adults are 1.21 times more likely to be overweight or obese compared to male adult households. Our study is consistent with a study that revealed that the probability of being overweight or obese is higher among women (47–49). This is explicitly related to the fact that, most of the time, overweight and obesity are caused by individuals having a propensity to overeat and engage in prolonged periods of inactivity. Therefore, frequent consumption of junk food and snacks, eating in a hurry, eating regularly, and a sedentary lifestyle are all potential causes of obesity and overweight in women (50–53).

Moreover, our study revealed that, according to this study, the place where adults reside was found to be statistically significant to outcome variables. Accordingly, those who reside in rural areas were 52% less likely to develop overweight or obesity than their counterparts. This finding is consistent with studies conducted in China (54, 55). One of the possible justifications related to this could be that those who reside in rural areas may have the opportunity to consume organic and healthy food, which inevitably results to them not being prone to overweight or obesity. Moreover, the majority of outdoor activities in which rural dwellers engage undeniably play a prominent role in burning calories, thereby hindering obesity or overweight (55, 56).

Furthermore, according to this study, marital status of adults was one of the independent predictors of obesity or overweight. Accordingly, the odds of developing obesity or overweight were 1.95 times higher among married adults than those of their counterparts. This is in harmony with the studies conducted in Greece (57), USA (58), and Iran (59). This could be explained by the fact that in culturally deep-rooted counties such as Ethiopia, married men probably tend to be socially resigned, less focused on being attractive, and less physically active, which explicitly contribute to developing obesity or overweight.

Finally, our study revealed that overweight and obesity distribution was varied across Ethiopian regions. Socioeconomic variables, including economy, eating habits, productivity, and environmental circumstances, are strongly correlated with obesity and overweight (60–62). Therefore, in our study setting, Ethiopia has different regions, which are vividly found in the categories of several economic, agricultural, and environmental variations that either directly or indirectly expose people to those various socioeconomic and cultural elements that resulted in different levels of overweight and obesity among regions.

### Practice implications

The findings of this study can be used to guide the development of programs aimed at preventing overweight and obesity in Ethiopia by informing policymakers and other stakeholders about this emerging nutrition-related problem among adult households in Ethiopia and also provide compelling evidence of the factors influencing overweight and obesity.

### Limitations of the study

The EDHS is a cross-sectional survey that limits its ability to establish cause-and-effect relationships between smoking and overweight or obesity. The EDHS relies on self-reported information that can be inaccurate due to social desirability bias or recall bias. While the EDHS aims to be nationally representative, there might be some groups underrepresented in the sample. This could limit the generalizability of the findings to the entire population in the study area. Moreover, BMI as a predictor of overweight/obesity is somewhat questionable since there are several studies with contradictory ideas. Thus, further studies using longitudinal data to assess the temporal relationship between independent variables and overweight or obesity would help to determine if these factors precede overweight/obesity or vice versa.

## Conclusion

The study revealed that approximately 8.5% and 2.9% of Ethiopian adults were overweight and obese, respectively. Factors including smoking, sex, marital status, educational status, wealth index, residence, and region were significantly associated with overweight and obesity among adult households. As a result, enhancing lifestyle modification interventions on overweight and obesity is enormous, and conducting more assessments to have more tangible and clear evidence concerning the factors influencing BMI utilizing more representative data from local to global would be important.

## Data Availability

The original contributions presented in the study are included in the article/supplementary material. Further inquiries can be directed to the corresponding authors.

## References

[1] WHO. Overweight and obesity: world health Organization (2021). Available online at: https://www.who.int/news-room/fact-sheets/detail/obesity-and-overweight (Accessed January 2023).

[2] WHO. Overweight and obesity (2018). Available online at: https://apps.who.int/nutrition/publications/overweight/en/index.html (Accessed January 2023).

[3] World Health Organization. (2022). Available online at: https://www.euro.who.int/en/health-topics/noncommunicable-diseases/obesity (Accessed June 29, 2021).

[4] CDC. Defining adult overweight & Obesity (2022). Available online at: https://www.cdc.gov/obesity/php/data-research/adult-obesity-prevalence-maps.html.

[5] Roser HRaM. Obesity - our world in data (2017). Available online at: https://ourworldindata.org/obesity (Accessed November 29, 2022).

[6] Hecker J Freijer K Hiligsmann M Evers SMAA. Burden of disease study of overweight and obesity; the societal impact in terms of cost-of-illness and health-related quality of life. BMC Public Health. (2022) 22:46. doi: 10.1186/s12889-021-12449-2 34996413 PMC8740868

[7] Afshin A Forouzanfar MH Reitsma MB Sur P Estep K Lee A. Health effects of overweight and obesity in 195 countries over 25 years. N Engl J Med. (2017) 377:13–27. doi: 10.1056/NEJMoa1614362 28604169 PMC5477817

[8] Lenz M Richter T Mühlhauser I. The morbidity and mortality associated with overweight and obesity in adulthood: a systematic review. Dtsch Arztebl Int. (2019) 106:641–8. doi: 10.3238/arztebl.2009.0641 PMC2770228 19890430

[9] Lung T Jan S Tan EJ Killedar A Hayes A. Impact of overweight, obesity and severe obesity on life expectancy of Australian adults. Int J Obes (Lond). (2019) 43:782–9. doi: 10.1038/s41366-018-0210-2 30283076

[10] Malkin JD Baid D Alsukait RF Alghaith T Alluhidan M Alabdulkarim H. The economic burden of overweight and obesity in Saudi Arabia. PloS One. (2022) 17:e0264993. doi: 10.1371/journal.pone.0264993 35259190 PMC8903282

[11] Obesity W. Economic-impact of Overweight and obesity in Ethiopia. (2019).

[12] Mahase E. Global cost of overweight and obesity will hit $4.32tn a year by 2035, report warns. BMJ. (2023) 380:523. doi: 10.1136/bmj.p523 36868577

[13] Grover SA Kaouache M Rempel P Joseph L Dawes M Lau DC. Years of life lost and healthy life-years lost from diabetes and cardiovascular disease in overweight and obese people: a modelling study. Lancet Diabetes Endocrinol. (2015) 3:114. doi: 10.1016/S2213-8587(14)70229-3 25483220

[14] CDC. Health effects of overweight and obesity (2022). Available online at: https://www.cdc.gov/healthyweight/effects.

[15] Perreault L Laferrère B. Overweight and obesity in adults: Health consequences (2022). Available online at: https://www.uptodate.com/contents/overweight-and-obesity-in-adults-health-consequences (Accessed February 14, 2024).

[16] NIH. Overweight and obesity causes and risk factors. (2018).

[17] Medcrave. Prevention and control strategies of overweight and obesity (2017). Available online at: https://medcraveonline.com/AOWMC/prevention.

[18] CDC. Strategies to prevent obesity and other chronic diseases. (2016).

[19] WHO. Global strategy on diet, physical activity and health. (2021).

[20] Kassie AM Abate BB Kassaw MW. Prevalence of overweight/obesity among the adult population in Ethiopia: a systematic review and meta-analysis. BMJ Open in British (2020). 10.1136/bmjopen-2020-039200 PMC7412611 32764091

[21] WHO. Obesity rising in Africa, WHO analysis finds (2022). Available online at: https://www.afro.who.int/news/obesity-rising-africa-who-analysis-finds (Accessed January 2023).

[22] WHO. Controlling the global obesity epidemic. World Health organization (2021). Available at: https://www.who.int/activities/controlling-the-global-obesity-epidemic.

[23] Dagne S Gelaw YA Abebe Z Wassie MM. Factors associated with overweight and obesity among adults in northeast Ethiopia: a cross-sectional study. Diabetes Metab syndrome obesity: Targets Ther. (2019) 12:391–9. doi: 10.2147/DMSO.S179699 PMC6434910 30962699

[24] Toselli S Zaccagni L Celenza F Albertini A Gualdi-Russo E. Risk factors of overweight and obesity among preschool children with different ethnic background. Endocrine. (2015) 49:717–25. doi: 10.1007/s12020-014-0479-4 25420642

[25] Wolde HF Gonete KA Akalu TY Baraki AG Lakew AM. Factors affecting neonatal mortality in the general population: evidence from the 2016 Ethiopian Demographic and Health Survey (EDHS)—multilevel analysis. BMC Res Notes. (2019) 12:610. doi: 10.1186/s13104-019-4668-3 31547855 PMC6757386

[26] Central statistical agency E Calverton M, USA. Ethiopia demographic and health survey. Addis Ababa, Ethiopia (2016).

[27] Organization WH. Global database on body mass index: BMI classification. World Health Organization (2006).

[28] Zbikowski SM Jack LM McClure JB Deprey M Javitz HS McAfee TA. Utilization of services in a randomized trial testing phone- and web-based interventions for smoking cessation. Nicotine tobacco Res. (2011) 13:319–27. doi: 10.1093/ntr/ntq257 PMC3082503 21330267

[29] Klesges RC Meyers AW Klesges LM La Vasque ME. Smoking, body weight, and their effects on smoking behavior: a comprehensive review of the literature. psychol bulletin. (1989) 106:204–30. doi: 10.1037/0033-2909.106.2.204 2678202

[30] Munafò MR Tilling K Ben-Shlomo Y. Smoking status and body mass index: a longitudinal study. Nicotine tobacco Res. (2009) 11:765–71. doi: 10.1093/ntr/ntp062 19443785

[31] Helvaci MR Altintas E Yalcin A Muftuoglu OE Abyad A Pocock L. Smoking may not prevent overweight or obesity. EC Clin Med Case Rep. (2023) 6:21–8.

[32] Dare S Mackay DF Pell JP. Relationship between smoking and obesity: a cross-sectional study of 499,504 middle-aged adults in the UK general population. PloS One. (2015) 10:e0123579. doi: 10.1371/journal.pone.0123579 25886648 PMC4401671

[33] Potter BK Pederson LL Chan SS Aubut J-AL Koval JJJN Research T. Does a relationship exist between body weight, concerns about weight, and smoking among adolescents? integration literature an emphasis gender. (2004) 6:397–425. doi: 10.1080/14622200410001696529 15203775

[34] Klesges RC Eck LH Isbell TR Fulliton W Hanson CL. Smoking status: effects on the dietary intake, physical activity, and body fat of adult men. J Am J Clin Nutr. (1990) 51:784–9. doi: 10.1093/ajcn/51.5.784 2333836

[35] Schumann A Hapke U Rumpf HJ Meyer C John U. The association between degree of nicotine dependence and other health behaviours. Findings from a German general population study. Eur J Public Health. (2001) 11:450–2. doi: 10.1093/eurpub/11.4.450 11766489

[36] Jeon HG Kim G Jeong HS So WY. Association between cigarette smoking and physical fitness level of Korean adults and the elderly. Healthcare (Basel Switzerland). (2021) 9:185. doi: 10.3390/healthcare9020185 33572288 PMC7914849

[37] Darebo T Mesfin A Gebremedhin S. Prevalence and factors associated with overweight and obesity among adults in Hawassa city, southern Ethiopia: a community based cross-sectional study. BMC Obes. (2019) 6:8. doi: 10.1186/s40608-019-0227-7 30867934 PMC6398229

[38] Dagne S Menber Y Petrucka P Wassihun Y. Prevalence and associated factors of abdominal obesity among the adult population in Woldia town, Northeast Ethiopia, 2020: Community-based cross-sectional study. PloS One. (2021) 16:e0247960. doi: 10.1371/journal.pone.0247960 33684157 PMC7939297

[39] Templin T Oliveira C Hashiguchi T Thomson B Dieleman J Bendavid E. The overweight and obesity transition from the wealthy to the poor in low- and middle-income countries: A survey of household data from 103 countries. PloS Med. (2019) 16:e1002968. doi: 10.1371/journal.pmed.1002968 31774821 PMC6880978

[40] Bishwajit G. Household wealth status and overweight and obesity among adult women in Bangladesh and Nepal. Obes Sci Pract. (2017) 3:185–92. doi: 10.1002/osp4.v3.2 PMC5478806 28706731

[41] Haregu TN Mohamed SF Muthuri S Khayeka-Wandabwa C Kyobutungi C. Body mass index and wealth index: positively correlated indicators of health and wealth inequalities in Nairobi slums. Glob Health Epidemiol Genom. (2018) 3:e11. doi: 10.1017/gheg.2018.10 30263135 PMC6152486

[42] Biswas T Garnett SP Pervin S Rawal LB. The prevalence of underweight, overweight and obesity in Bangladeshi adults: Data from a national survey. PloS One. (2017) 12:e0177395. doi: 10.1371/journal.pone.0177395 28510585 PMC5433717

[43] Abrha S Shiferaw S Ahmed KY. Overweight and obesity and its socio-demographic correlates among urban Ethiopian women: evidence from the 2011 EDHS. BMC Public Health. (2016) 16:1–7. doi: 10.1186/s12889-016-3315-3 27457223 PMC4960736

[44] Nagata JM Garber AK Tabler JL Murray SB Bibbins-Domingo K. Prevalence and correlates of disordered eating behaviors among young adults with overweight or obesity. J Gen Internal Med. (2018) 33:1337–43. doi: 10.1007/s11606-018-4465-z PMC6082209 29948810

[45] Pagliai G Dinu M Madarena M Bonaccio M Iacoviello L Sofi F. Consumption of ultra-processed foods and health status: a systematic review and meta-analysis. Br J Nutr. (2021) 125:308–18. doi: 10.1017/S0007114520002688 PMC7844609 32792031

[46] Wang L Zhou B Zhao Z Yang L Zhang M Jiang Y. Body-mass index and obesity in urban and rural China: findings from consecutive nationally representative surveys during 2004–18. Lancet. (2021) 398:53–63. doi: 10.1016/S0140-6736(21)00798-4 34217401 PMC7617101

[47] Rachmi C Li M Baur LA. Overweight and obesity in Indonesia: prevalence and risk factors—a literature review. Public Health. (2017) 147:20–9. doi: 10.1016/j.puhe.2017.02.002 28404492

[48] Alqarni SSM. A review of prevalence of obesity in Saudi Arabia. J Obes Eat Disord. (2016) 2:1–6. doi: 10.21767/2471-8203.100025

[49] Mannan M Mamun A Doi S Clavarino A. Prospective associations between depression and obesity for adolescent males and females-a systematic review and meta-analysis of longitudinal studies. PloS One. (2016) 11:e0157240. doi: 10.1371/journal.pone.0157240 27285386 PMC4902254

[50] health i. Why is Obesity Becoming Prevalent in Women. (2022).

[51] Kulie T Slattengren A Redmer J Counts H Eglash A Schrager S. Obesity and women's health: an evidence-based review. J Am Board Fam Med. (2011) 24:75–85. doi: 10.3122/jabfm.2011.01.100076 21209347

[52] Kapoor N Arora S Kalra S. Gender disparities in people living with obesity - an unchartered territory. J Midlife Health. (2021) 12:103–7. doi: 10.4103/jmh.jmh_48_21 PMC8409720 34526743

[53] health I. Why is Obesity Becoming Prevalent in Women? (2022). Available online at: https://www.nhlbi.nih.gov/health/overweight-and-obesity/women (Accessed August 12, 2023).

[54] Yan Z Ronghua Z Biao Z Lichun H Jiang C Fang G. A comparison study on the prevalence of obesity and its associated factors among city, township and rural area adults in China. BMJ Open. (2015) 5:e008417. doi: 10.1136/bmjopen-2015-008417 PMC4513451 26179650

[55] Taylor R Badcock J King H Pargeter K Zimmet P Fred T. Dietary intake, exercise, obesity and noncommunicable disease in rural and urban populations of three Pacific Island countries. J Am Coll Nutr (1992) 11(3):283–93. doi: 10.1080/07315724.1992.10718229 1619180

[56] Du H Bennett D Li L Whitlock G Guo Y Collins R. China Kadoorie Biobank Collaborative Group Physical activity and sedentary leisure time and their associations with BMI, waist circumference, and percentage body fat in 0.5 million adults: the China Kadoorie Biobank study. Am J Clin Nutr (2013) 97(3):487–96. doi: 10.3945/ajcn.112.046854 PMC4345799 23364014

[57] Tzotzas T Vlahavas G Papadopoulou SK Kapantais E Kaklamanou D Hassapidou M. Marital status and educational level associated to obesity in Greek adults: data from the National Epidemiological Survey. BMC Public Health. (2010) 10:732. doi: 10.1186/1471-2458-10-732 21110843 PMC3004837

[58] Molarius A Seidell JC Sans S Tuomilehto J Kuulasmaa K. Educational level, relative body weight, and changes in their association over 10 years: an international perspective from the WHO MONICA Project. J Am J Public Health. (2000) 90:1260. doi: 10.2105/AJPH.90.8.1260 10937007 PMC1446346

[59] Janghorbani M Amini M Rezvanian H Gouya MM Delavari A Alikhani S. Association of body mass index and abdominal obesity with marital status in adults. Arch Iranian Med. (2008) 11:274–81. 18426318

[60] Qasim A Turcotte M De Souza R Samaan M Champredon D Dushoff J. On the origin of obesity: identifying the biological, environmental and cultural drivers of genetic risk among human populations. Obes Rev. (2018) 19:121–49. doi: 10.1111/obr.12625 29144594

[61] Silventoinen K Jelenkovic A Sund R Yokoyama Y Hur Y-M Cozen W. Differences in genetic and environmental variation in adult BMI by sex, age, time period, and region: an individual-based pooled analysis of 40 twin cohorts. Am J Clin Nutr. (2017) 106:457–66. doi: 10.3945/ajcn.117.153643 PMC5525120 28679550

[62] Burgess E Hassmén P Pumpa KL. Determinants of adherence to lifestyle intervention in adults with obesity: a systematic review. Clin Obes. (2017) 7:123–35. doi: 10.1111/cob.2017.7.issue-3 28296261

